# Molecular Mechanisms of *Acanthamoeba castellanii* Response to Different Sources of Oxidative Stress

**DOI:** 10.1021/acs.jproteome.4c00573

**Published:** 2025-01-20

**Authors:** Kateřina Ženíšková, Pavel Stopka, Tania Martín-Pérez, Guillaume Chevreux, Maria Grechnikova, Eliška Drncová, Ronald Malych, Jan Mach, Julia Walochnik, Jean-Michel Camadro, Robert Sutak

**Affiliations:** † Department of Parasitology, Faculty of Science, BIOCEV, Charles University, Vestec 25250, Czech Republic; ‡ Department of Zoology, Faculty of Science, BIOCEV, Charles University, Vestec 25250, Czech Republic; § Center for Pathophysiology, Infectiology and Immunology, Institute of Specific Prophylaxis and Tropical Medicine, Medical University of Vienna, Vienna 1090, Austria; ∥ Université de Paris Cité, CNRS, Institut Jacques Monod, Paris F-75013, France

**Keywords:** Acanthamoeba, ROS, proteomics, ABC transporter, oxidative stress

## Abstract

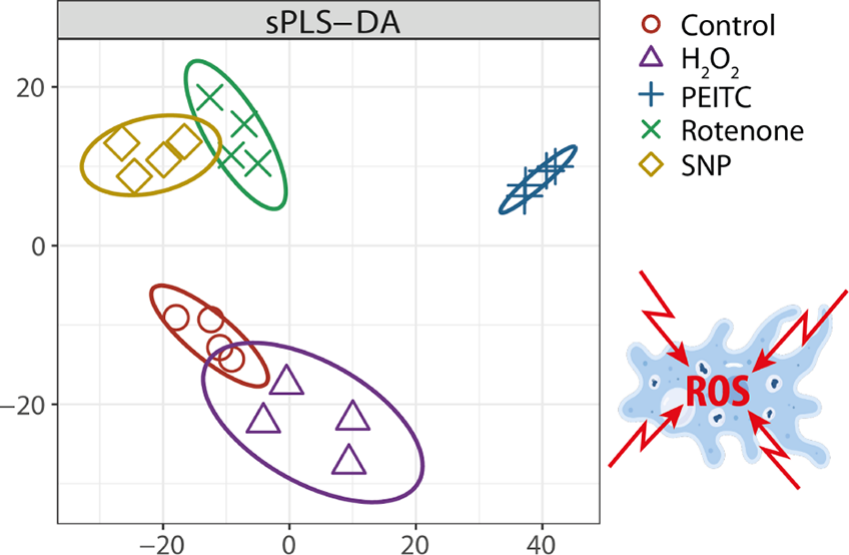

Oxidative stress is a biological principle affecting all life on Earth and is also an important factor in the pathogen-host relationship. The pathogenic free-living amoeba *Acanthamoeba castellanii* has several pathways to cope with reactive oxygen species and the damage that they cause. In this study, we aimed to provide a comprehensive analysis of the amoeba’s response to different sources of oxidative stress. Using whole-cell proteomic analysis, we obtained a complex picture of the changes in the proteome and identified potential key players in the defense against oxidative stress. Importantly, from the differential proteomics analysis, we identified a candidate efflux pump that may be involved in *Acanthamoeba* drug resistance.

## 1 Introduction

Reactive oxygen species (ROS), such as superoxide radical (•O^2–^), singlet oxygen (^1^O_2_), hydroxyl radical (•OH), and hydrogen peroxide (H_2_O_2_), are highly reactive molecules with unpaired valence electrons or unstable bonds that are produced as byproducts of aerobic metabolism.^1^ Multiple enzymes or intracellular chemicals are able to cope with excess ROS. However, imbalances in ROS homeostasis can lead to oxidative stress, which is associated with aging^2^ and various diseases, including cancer,^3−5^ inflammatory diseases, and neurological disorders.^6−9^ However, ROS also play several beneficial roles when present at moderate levels. They act as intracellular signaling molecules,^10,11^ participate in hormonal biosynthesis,^12^ and contribute to immune responses by exhibiting antimicrobial activity. Under physiological conditions, the cellular ROS level is maintained by dynamic equilibrium, balanced by several mechanisms of constant ROS production and elimination.

In excess, ROS cause oxidative modifications of all major cellular macromolecules, such as lipids, proteins, DNA, and carbohydrates, leading to alteration of their biological function, increasing mutagenesis, and finally leading to cell death.^13,14^ Due to their toxic properties, these highly reactive molecules are used as host antimicrobial strategies against a variety of pathogens.^15,16^ An arsenal of host immune cells (neutrophils and macrophages) phagocytose pathogens and trigger an oxidative burst—the rapid induction and release of ROS molecules—against exogenous pathogens.^16−19^ Pathogens, however, employ a variety of mechanisms to counter and evade the immune response using different strategies such as enhancing antioxidant/ROS-detoxification pathways. Investigating the pathogen’s ability to face oxidative stress by identifying its key players in ROS-detoxification pathways may shed light on the pathogen’s survival strategy and pathomechanism and potentially lead to the development of new therapeutic options.

This study focuses on *Acanthamoeba castellanii*, a free-living amoeba that occupies diverse habitats such as soil or water environments,^20^ but also causes partly severe infections in humans. On the one hand, it is the causative agent of *Acanthamoeba* keratitis,^21,22^ a rare but serious ocular infection that can lead to visual loss. On the other hand, it can cause granulomatous amebic encephalitis, an often fatal brain and spinal cord infection that typically occurs in immunocompromised individuals.^23,24^

The fact that this organism can both live freely and become an opportunistic pathogen, causing two very different diseases in humans, reinforces the fact that *A. castellanii*possesses a remarkable ability to adapt to considerable environmental changes. It has a number of mechanisms that protect it from oxidative stress, including mitochondrial energy-dissipating systems,^25^ catalase,^26^ superoxide dismutase,^27^ and both thioredoxin and glutathione systems.^28^ Recently, oxidative stress-induced transcriptional changes of key enzymes involved in the thioredoxin and glutathione systems of *A. castellanii* have been described, highlighting the complexity of the amoeba’s redox system.^29^

The aim of this study was to investigate the ability of *A. castellanii* to counteract the oxidative stress induced by various ROS-inducing agents. Proteomic analysis, together with RT-qPCR, was used to obtain a comprehensive view of the response to these challenging conditions, with the ultimate goal of identifying *A. castellanii* key players in the defense against oxidative stress.

## 2 Methods

### 2.1 Growth Analysis

*A. castellanii* cells, strain Neff (ATCC 30010) (1 × 10^5^ cells) were cultivated in a 12-well plate aerobic culture flask at 27 °C in PYG medium (0.75% yeast extract, 0.75% proteose peptone, and 1.5% glucose)^30^ supplemented with different concentrations of various ROS-inducing agents: sodium nitroprusside (SNP) (Merck, USA) (10 μM; 100 μM; 1 mM), H_2_O_2_ (10 μM; 100 μM; 250 μM; 1 mM), phenethyl isothiocyanate (PEITC) (Merck, USA) (6.25 μM; 12.5 μM; 15 μM; 25 μM; 50 μM), or rotenone (Merck, USA) (25 μM; 50 μM). Cells supplemented with 0.45% or 1% ethanol, respectively, were used as a control. Cell density was measured every 24 h for 3 days (except for the first 19-hour time point) using a GUAVA EasyCyte 8HT flow cytometer (Merck, USA) after fixation with 2% paraformaldehyde.

### 2.2 Oxyblot

Oxidative damage to proteins caused by selected ROS-inducing agents was determined by immunoblot detection of carbonyl groups using the OxyBlot protein oxidation detection kit (Merck Millipore, USA) according to the manufacturer’s protocol. Briefly, 20 μg of protein sample in PBS was treated with 6% SDS and incubated with 2,4-dinitrophenylhydrazine. The dinitrophenylhydrazone-derivative residues were detected by a specific primary antibody in conjunction with a secondary antibody (provided in the kit) and visualized using the enhanced chemiluminescence system Amersham Imager 600 (GE Healthcare Life Sciences, USA).

### 2.3 Sample Preparation for LC-MS/MS

*A. castellanii* cells were grown in 25 cm^2^ aerobic culture flasks at 27 °C in PYG medium supplemented with 100 μM SNP, 250 μM H_2_O_2_, 6.25 μM PEITC, or 50 μM Rotenone, respectively, for 2 or 8 h, in four biological replicates. After the incubation, cells were washed twice (1200 g, 10 min, 4 °C) with phosphate-buffered saline (PBS) containing a protease inhibitor cocktail (Merck, USA), phosphatase cocktail II+III, and components to preserve the acetylation state of proteins: 40 μM Trichostatin A, 1 mM EX-527, 400 mM Nicotinamide, and 200 mM Sodium Butyrate (all Merck, USA). Pellets were then resuspended in RIPA buffer (ThermoFisher, USA) containing the respective inhibitors (described above). Subsequently, the samples were vigorously pipetted in and out to ensure cell lysis, followed by centrifugation at 14,000*g* for 15 min at 4 °C. The resulting supernatant was carefully transferred to a fresh tube, and the protein concentration of the samples was determined using the BCA kit (Sigma-Aldrich, USA). The samples were stored at −80 °C until their next use.

### 2.4 Samples Preparation for Proteomic Analysis

Six times the volume of cooled acetone (−20 °C) was added to the sample volume containing 10 μg of protein extracts. The vortexed tubes were incubated overnight at −20 °C and then centrifuged for 10 min at 11,000 rpm and 4 °C. The protein pellets were dissolved in buffer (8 M urea; 25 mM NH_4_HCO_3_). The samples were then digested overnight at 37 °C by sequencing grade trypsin (enzyme:sample ratio 1:20; Promega, USA). The digested peptides were loaded and desalted on Evotips (Evosep One, Denmark) according to the manufacturer’s instructions.

### 2.5 LC-MS/MS Analysis

Samples were analyzed on a timsTOF Pro 2 mass spectrometer (Bruker Daltonics, Germany) coupled to an Evosep One system (Evosep, Denmark) operating with the 30 samples/day method developed by the manufacturer. Chemicals for the method, MS-grade Acetonitrile (ACN), H_2_O and formic acid (FA) were from Thermo Chemical (USA). Briefly, the method is based on a 44-minute gradient and a total cycle time of 48 min with a C18 analytical column (0.15 × 150 mm, 1.9 μm beads, ref EV-1106, Evosep, Denmark) equilibrated at 40 °C and operated at a flow rate of 500 nL/min. H_2_O/0.1% FA was used as solvent A and ACN/0.1% FA as solvent B. The timsTOF Pro 2 was operated in DDA PASEF (Data-Dependent Acquisition that uses Parallel Accumulation Serial Fragmentation) mode over a 1.3 s cycle time. Mass spectra for MS and MS/MS scans were recorded between 100 and 1,700 *m*/*z*. The mass spectrometry proteomics data have been deposited to the ProteomeXchange Consortium via the PRIDE^31^ partner repository with the dataset identifier PXD052473.

### 2.6 Data Analysis from LC-MS/MS

Peak Online X software (build 1.6, Bioinformatics Solutions Inc.) was used to search for proteins against the *Acanthamoeba castellanii* database (UniProt release 2022_01, 14979 entries). The parent mass tolerance was set to 20 ppm with a fragment mass tolerance of 0.05 Da. Semispecific tryptic cleavage was selected, and a maximum of 2 missed cleavages was allowed. Half disulfide bridge (C) was set as a fixed modification. Oxidation (M) and deamidation (NQ) were set as possible variable modifications. The maximum number of variable modifications per peptide was limited to 3. Identifications were filtered based on a 1% false discovery rate (FDR) threshold at both the peptide and protein group levels. Another search was performed to screen the oxidative status of cysteines. In this search, half disulfide bridge (C), cysteine oxidation to cysteic acid (C), cysteinylation (C), glutathione disulfide (C), oxidation or hydroxylation (C), dihydroxylation (C), oxidation (M), and deamidation (NQ) were all set as variable modifications. Protein identifications were only considered if at least two unique identified peptides were present within a single protein. Multivariate statistics on protein measurements were performed using Qlucore Omics Explorer 3.7 (Qlucore AB, SWEDEN). A positive threshold of 1 was set to allow a log2 transformation of abundance data for normalization; i.e., all abundance data values below the threshold are replaced by 1 before transformation. The transformed data were finally used for statistical analysis, i.e., the evaluation of differentially present proteins between two groups using a bilateral Student’s *t*-test and assuming equal variance between groups. A *p*-value better than 0.05 was used to filter out differential candidates.

### 2.7 Western Blot

To confirm the results of the proteomic analysis, the native expression of thioredoxin reductase (ACA1_398900) was visualized using a purified rabbit polyclonal antibody.^28^ SDS-PAGE and Western blotting were performed according to standard protocols in a Mini Protean Tetra Cell (Bio-Rad, USA). Blots were developed with peroxidase-conjugated goat antirabbit secondary antibody (A9169, Merck, USA). The signal was detected on an Amersham Imager 600 (GE Healthcare Life Sciences, USA) using an Immobilon Forte Western HRP substrate (Merck, USA).

### 2.8 PCR Primer Efficiency Study

Two pairs of primers for each gene of interest (GOI) were designed using Primer3 and initially tested in conventional PCR using genomic DNA. All primers were synthesized by Microsynth. Then, standard curves were generated with 5 points of 10-fold serial dilutions of RNA to calculate the primer efficiency (E) and the correlation coefficients (R²). Efficiency was calculated according to the formula *E* = (10^–1^/slope −1)*100. The primer pair with better efficiency in RT-qPCR was selected for further experiments (Table 1).

**Table 1:** Primer Details of the Gene of Interest

Primer	Primer sequence (5′–3′)	Amplicon length (bp)	Average T_m_ (°C)	Amplification efficiency (%)	GenBank accession no.
OR1	CCAAGATCGTTCGCTTCCAT	103	63.5	112.26	XM_004333761.1 ACA1_362830
	CTTCACTTGGAGCCTGACCT		64.7		
OR2	CAAGATCGCCAAGACCTTCC	92	3.7	106.72	
	AGCGAGATGACCACTTTGCC		65.7		
GST1	CAGTTTGGGTCGCAGGTTAC	95	64.1	129.28	XM_004343605.1 ACA1_099220
	CTTCTTGGTGAGCTCCTCCT		64.2		
GST2	CCAGCTCATCAAGAACCAAGAC	146	64.1	102.05	
	ACCAAAGAGAACGACTCGCC		65.3		
PHO1	CCACCAAGTTCAACATGCGA	89	64.1	105.72	XM_004339082.1 ACA1_057530
	GACTGTAGCACATCTCGGGA		64.1		
PHO2	AAGAGGACGACGAGGACTAC	75	63.4	108.97	
	GCCTTCTTCTCACGCTCAAC		64.1		

### 2.9 RNA Extraction and Quantitative Real-Time PCR (RT-qPCR)

The RNA was isolated using an innuPREP RNA Mini Kit 2.0 (Analytik Jena, Germany) following the manufacturer’s protocol. The concentration and purity of RNA were measured with a NanoDrop spectrophotometer ND1000 (NanoDrop Technologies, USA). All RNA samples were diluted to 10 ng/μL using nuclease-free water and stored at −80 °C until use.

RT-qPCR was performed in a CFX96 thermocycler (Bio-Rad, USA) using the Luna Universal One-Step RT-qPCR kit (E3005L, New England BioLabs, USA). The reaction mixture (20 μL per reaction) contained 10 μL of Luna Universal One-Step Reaction Mix 2x, 1 μL of Luna WarmStart RT Enzyme Mix 20x, 400 nM of each primer, and 50 ng of RNA (5 μL of 10 ng/μL). The RT-qPCR profile included a reverse transcription step at 55 °C for 10 min, an initial denaturation step at 95 °C for 1 min, followed by 40 cycles of denaturation at 95 °C for 10 s and extension at 60 °C for 60 s, and a melting curve was performed at the end of the run by stepwise (0.5 °C per 5 s) increasing the temperature from 60 to 95 °C. All experiments were carried out in two technical and three biological replicates. The relative expression of target genes was normalized using the formula described by^32^ and as reference genes (RG) the 18S rRNA gene and the hypoxanthine-guanine phosphoribosyltransferase (HPRT).^33^
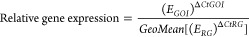


Statistical analysis was performed with GraphPad Prism 9 (GraphPad Software Inc., USA). To determine statistical significance among investigated groups, one-way analysis of variance (ANOVA) was performed. A statistical difference was considered significant when *p* < 0.05.

### 2.10 ABC Transporter Localization

Gene was subcloned into the pTN plasmid, which allows expression of N-terminally GFP-tagged proteins^30^ and transfected into the *A. castellanii* cells according to the published protocol (dx.doi.org/10.17504/protocols.io.s4regv6). Live cell microscopy was done to visualize the GFP signal using a Leica TCS SP8 WLL SMD-FLIM microscope (Leica, Germany) equipped with an HC PL APO CS2 63*x*/1.20 water objective (excitation 488 nm, emission 498–551 nm).^30^ Acquired pictures were processed using Fiji software.^34^

## 3 Results

### 3.1 A Comparative Analysis of Proteome Responses to Various ROS-Inducing Agents

**Figure 1:**
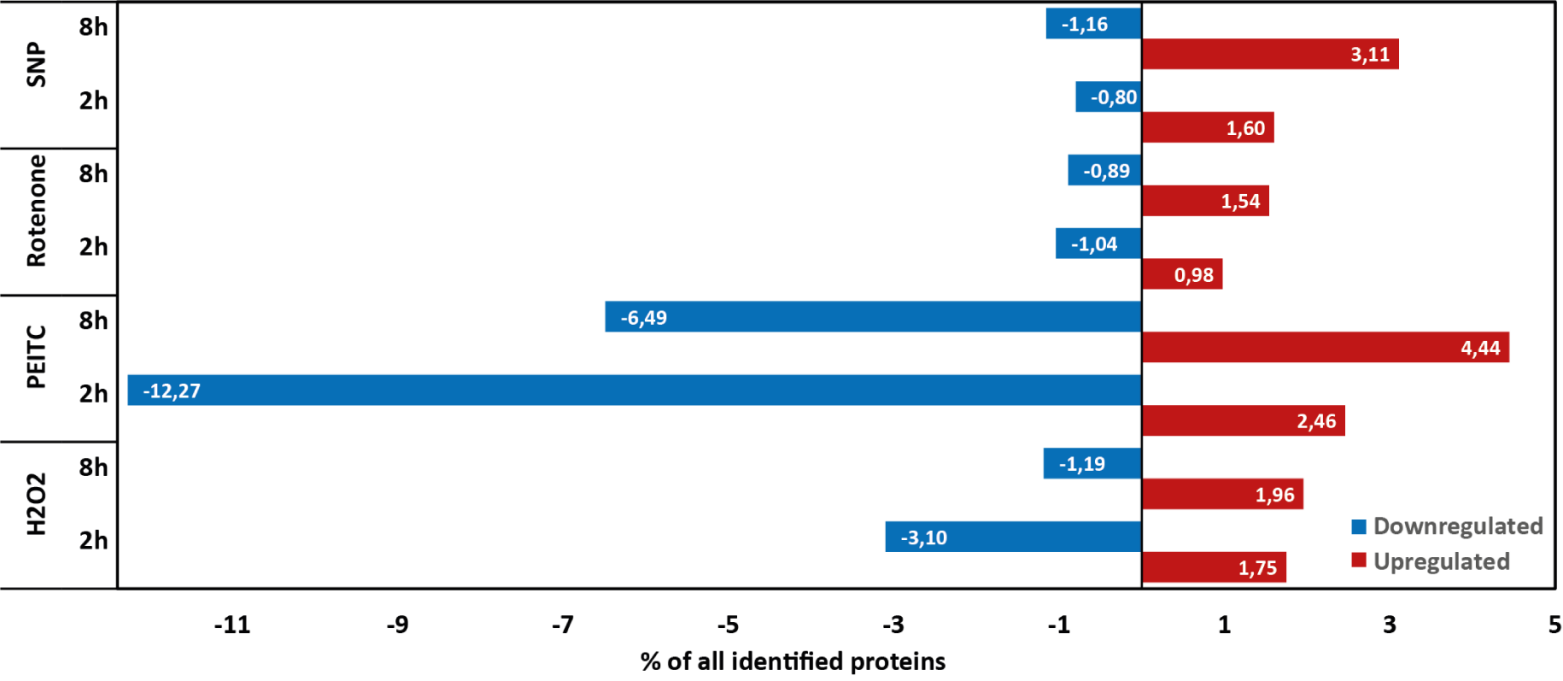
Protein-level response to selected ROS-inducing agents in *A. castellanii*. Significantly upregulated proteins (shown in red) or downregulated proteins (shown in blue) are visualized as a percentage of all identified proteins in the proteomic analysis (3375). All proteins meeting criteria for up-regulated or downregulated proteins (criteria indicated in section 2.6) are listed in Table S1.

Label-free proteomic analysis was employed to investigate the response of *A. castellanii* to different conditions of ROS induction at the protein level. Several known ROS-inducing agents were selected to induce oxidative stress in the cells: H_2_O_2_, phenethylisothiocyanate (PEITC),^35−37^ and rotenone,^38,39^ as well as a source of both nitrosative and oxidative stress: sodium nitroprusside (SNP).^40,41^ The concentrations of the agents were selected based on the growth analysis (see Figure S1), and the ability of these agents to induce oxidative damage to proteins of *A. castellanii* at selected concentrations was confirmed using the Oxyblot Protein Oxidation Detection Kit (Figure S3). Two incubation points of 2 and 8 h were set to determine the immediate and prolonged response of the *A. castellanii* proteome to selected conditions. Overall, 3,375 proteins were identified by label-free proteomic analysis. Figure 1 visualizes the differential proteomic response to selected ROS-inducing agents, where the amount of upregulated and downregulated proteins in each condition is shown as a percentage, with the total amount of proteins identified in the proteomic analysis considered as 100%. The number of identified proteins under different conditions exhibited minimal variation, with a difference of less than 5%. Additionally, fewer than 15 proteins were unique to each condition. The highest number of significantly changed proteins was identified under PEITC conditions at both incubation time points, whereas the lowest total number of significantly changed proteins was identified in rotenone treatment.

Changes in the proteome were evident as early as two h of incubation with each agent, and the number of proteins with increased levels was greater after 8 h in all conditions, with the lowest change in H_2_O_2_. On the other hand, the number of proteins with reduced levels was lower after 8 h than after 2 h, except for incubation with SNP. To validate the observed changes, a specific antibody against thioredoxin reductase (TrxR-S, ACA1_398900) was used to confirm its increased expression in cells incubated in the presence of H_2_O_2_ by Western blot (Figure S4).

#### 3.1.1 Candidate Gene Approach Analysis of Proteomic Data

**Figure 2:**
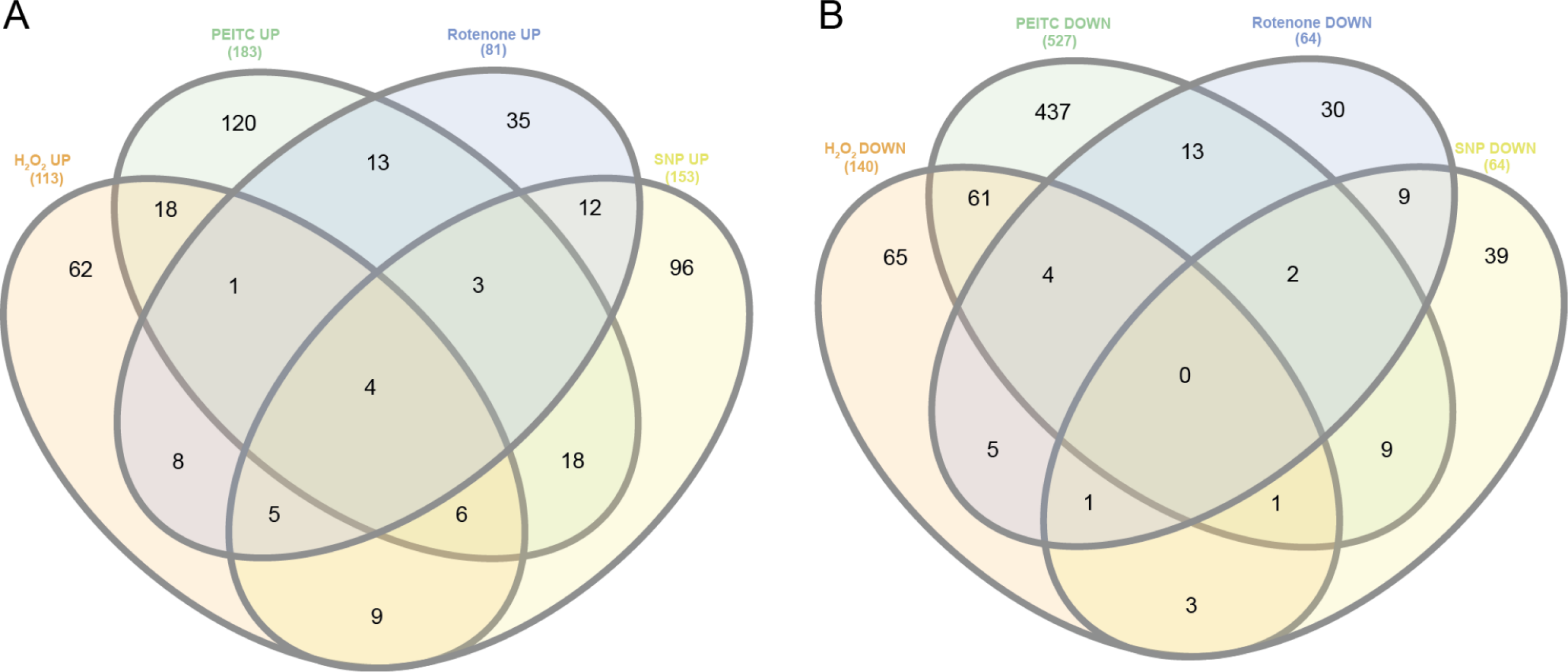
Venn diagrams created by using http://www.interactivenn.net/(42) showing the number of unique and common identified proteins between conditions in diagram (A) from the total number of significantly upregulated proteins (531) and in diagram (B) from the total number of significantly downregulated proteins (795) in all the ROS inducing conditions (Table S1).

In order to explore the cellular response to oxidative stress that is common to all studied conditions, we created a Venn diagram from the list of significantly upregulated proteins (531) (Figures 2 and S2). Four proteins: oxidoreductase (ACA1_362830), NADPH-dependent FMN reductase (ACA1_175790), a protein from the glutathione transferase family (ACA1_099220), and phosphatase (ACA1_057530) had elevated levels under all selected ROS-inducing conditions after 2 and/or 8 h of incubation, suggesting their key role in coping with oxidative stress in *A. castellanii* (Table 2). No protein showed reduced levels in all four conditions, and, in general, the overlap of downregulated proteins between the different conditions was smaller than that of upregulated proteins. We also analyzed the effect of oxidative stress on post-translational cysteine modifications, which, consistent with the upregulation of numerous glutathione transferases, are indeed affected with the highest changes after SNP (at 8 h) and PEITC treatment (Table S2).

**Table 2:** List of Proteins with Significantly Elevated Expression in all ROS-inducing Conditions Based on the Venn Diagram (Figure 2)

ACA1_362830	Oxidoreductase, putative
ACA1_175790	NADPH-dependent FMN reductase domain containing protein
ACA1_099220	glutathione transferase family protein
ACA1_057530	phosphatase, putative

### 3.2 Correlation between Gene Expression and Protein Level Changes

**Figure 3:**
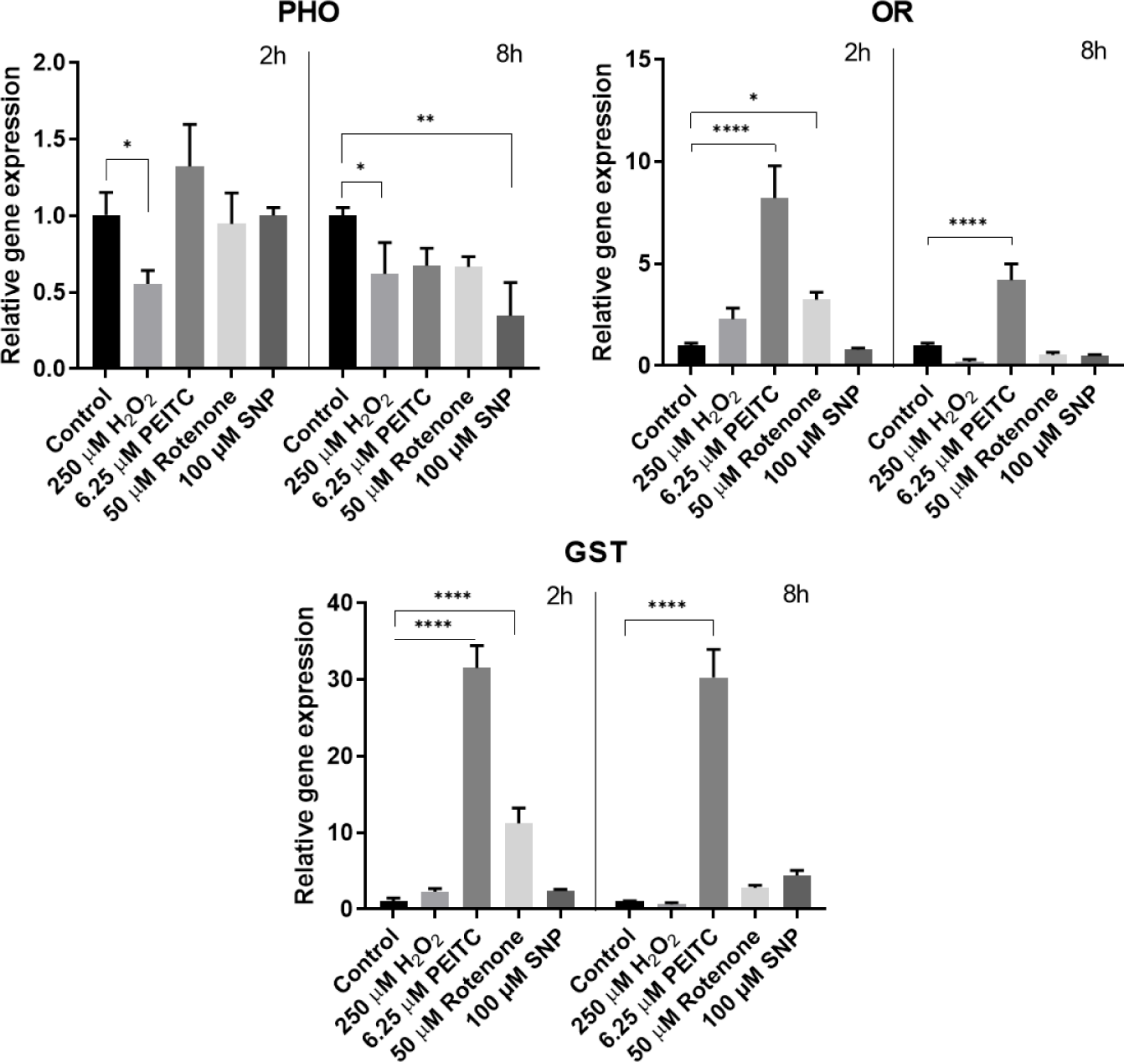
Relative gene expression of the genes of interest after 2 and 8 h of treatment with 250 μM H_2_O_2_, 6.25 μM PEITC, 50 μM Rotenone, and 100 μM SNP (mean ± s.d., *n* = 3). *P*-values are represented with asterisks as follows: * for ≤0.05, ** for ≤0.01, *** for ≤0.001, and **** for <0.0001.

To determine whether the increase in protein levels under oxidative stress occurs at the level of gene expression, we performed RT-qPCR with 3 selected proteins affected in all conditions. The relative gene expression with different treatments at 2 and 8 h is shown in Figure 3. After 2 h, the relative expression of oxidoreductase (OR) and glutathione transferase (GST) increased after treatment with PEITC and rotenone, while the effect was lower with the latter compound. There was no increase in the relative expression of phosphatase (PHO). Interestingly, the relative expression obtained for the three GOIs was generally lower after 8 h of treatment and the increase of OR and GST was significant only after treatment with PEITC. Similar to the 2-h treatment, no increase in the relative expression of PHO was observed after 8 h.

### 3.3 ABC Transporter

**Figure 4:**
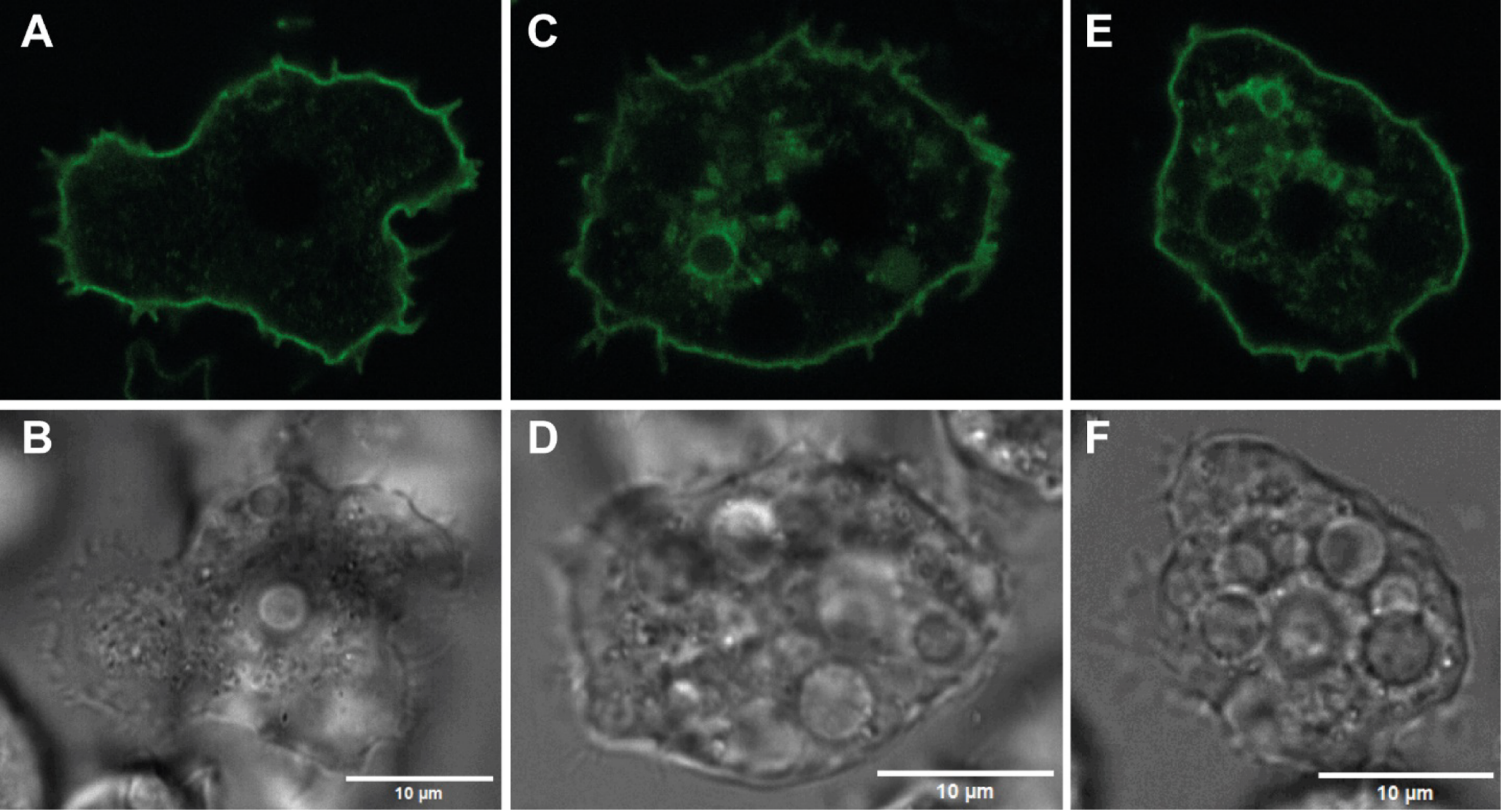
Live cell microscopy of GFP-tagged *A. castellanii* ABC transporter (A–E) and differential interference contrast (B–F) shows plasmatic membrane localization.

Among the most affected proteins, we identified an ABC transporter (ACA1_352460) that was up-regulated upon treatment with rotenone and PEITC, and whose levels were elevated after both 2 and 8 h of incubation. Members of this family of proteins in eukaryotes are mostly effluxers. Analysis using the HHpred tool clearly predicts that the amoeba homologue is a pleiotropic drug resistance protein, and because of the importance of this family of proteins in microbial drug resistance^43^ we decided to focus further on this transporter. To support the hypothesis that it is a cellular efflux transporter, we determined its cellular localization by expressing it with a GFP tag. As shown in Figure 4, the protein is mainly localized to the plasma membrane, as expected.

### 3.4 Sparse Partial Least-Squares Discriminant Analysis of Proteomic Data

**Figure 5:**
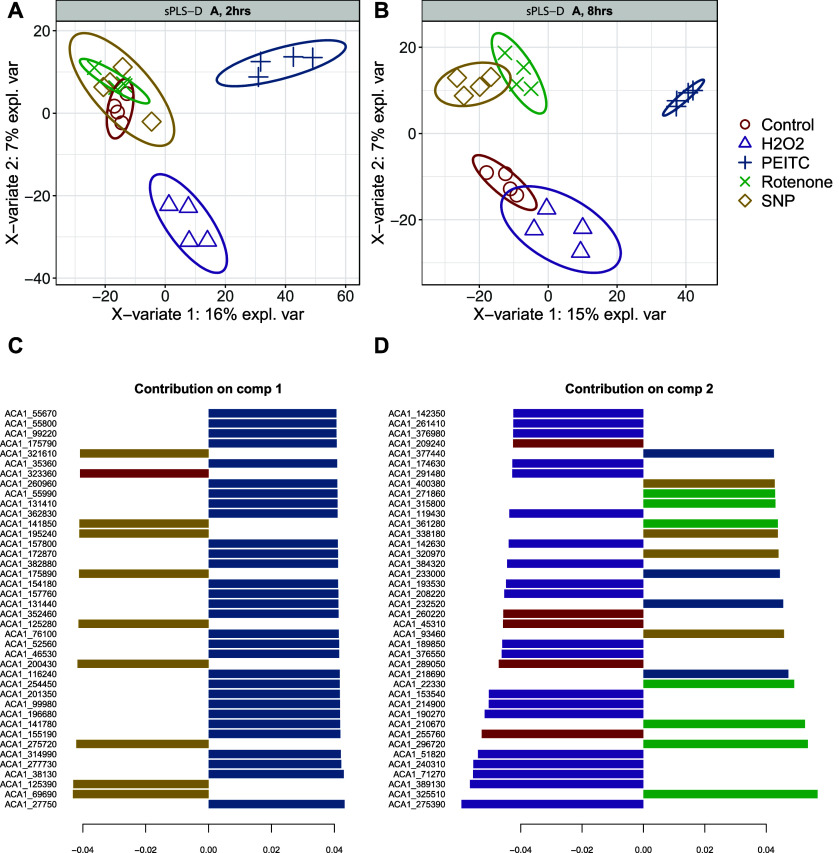
Sparse Partial Least Squares Discriminant Analysis (sPLS-DA). The results show that the proteomes after 2 h of cultivation generate only small changes (A) while 8 h of cultivation yields extensive changes in phenotype when compared to controls, B. Loading values in the 1st (C) and 2nd dimensions (D) there are at least three proteins detected in previous analyses and are also responsible for the differentiation (PEITC), but there are many other proteins that also differentiate given types of cultivation.

Next, we aimed to identify the most predictive and discriminative features in our data in order to classify the samples. This step is essential to determine whether upregulated proteins in our data set show systematic features along with other proteins or whether they show this trend simply by chance. Thus, following the candidate-gene approach, we aimed to search for patterns on a global scale and whether the detected proteins above can be corroborated using sPLS-DA. First, we normalized data with normalyzerDE^44^ and from all the performed normalizations, we selected VSN normalization, which provided the lowest within-group variation. Next, we used Sparse Partial Least Squares Discriminant Analysis (sPLS-DA) and Area Under Curve Analysis (AUC) to find potential sources of variation in our data. AUC analysis is based on the selectivity and specificity of sPLS-DA and represents the probability that the sPLS-DA model will rank the positive examples higher than the negative examples. In Figure 5A, we clearly see that, after 2 h of cultivation, PEITC had a strong influence upon separation from controls (*X*-axis, AUC = 1,

*p* = 0.002), and similarly, H_2_O_2_ samples diverged from controls (*Y*-axis, AUC = 1, *p* = 0.02). However, Rotenone and SNP overlapped with controls. After 8 h of cultivation, all conditions significantly diverged from controls in either the first or second dimension. For example, in the first component (*X*-axis) PEITC significantly diverges from the others (AUC = 1, *p* = 0.0025), while the second component (*Y*-axis) discriminates H_2_O_2_ from controls and all other conditions (AUC = 1, *p* = 0.0025), as well as PEITC (AUC = 1, *p* = 0.0025), SNP (AUC = 0.97, *p* = 0.005), and Rotenone (AUC = 0.84, *p* = 0.04), which are above zero on the *Y*-axis Figure 5B.

To find out the biological relevance of these changes, we extracted loadings from sPLS-DA (Figure 5C,D) and interestingly, three of the previously mentioned proteins (ACA1_175790, ACA1_362830, ACA1_352460) responsible for the differentiation of PEITC samples were also detected (Figure 5B and are highlighted in Figure 5C. This is additional evidence that these proteins are markers of oxidative stress mentioned above. On a global scale, however, there is a detectable small effect of many proteins in combination rather than a strong effect of a few. For example, ACA1_325510 (actin bundling protein), ACA1_296720 (histone deacetylase 1, putative), and ACA1_210670 (universal stress domain containing protein) contribute to the separation of Rotenone samples (2nd comp. on the *Y*-axis, Figure 5D). Similarly, in PEITC samples, separation in *Y*-axis of PEITC samples is driven by ACA1_218690 (1,2-dihydroxy-3-keto-5-methylthiopentene dioxygenase), which is annotated as an enzyme that catalyzes two different reactions between oxygen and the acireductone and depends upon the metal bound in the active site. In addition, this analysis shows that the overall phenotype changes at the proteome level and not just selected proteins under the four types of oxidative stress.

## 4 Discussion

Understanding the cellular response to oxidative stress is of particular importance in parasites, as they encounter oxidative stress during host invasion. To gain a broad insight into the mechanisms by which *Acanthamoeba* combats oxidative stress, we used four different sources for its induction: H_2_O_2_, as the most direct and commonly used generator of oxidative stress/damage; rotenone and PEITC, which cause it indirectly through more complex/metabolic mechanisms; and SNP, which induces nitrosative stress, closely related to oxidative stress.^45^ The most significant changes were observed after treatment with PEITC, suggesting a complex effect on the cell. The changes were more pronounced after 8 h of treatment, except for H_2_O_2_, probably due to its instability. The overlap of downregulated proteins between the different conditions was less evident than that of upregulated proteins, indicating that the specific response to oxidative stress is more directed toward upregulation of defense pathways, and the decrease in protein levels is more the result of their degradation, disrupted metabolism, and decreased cellular fitness. Correlations between proteomic data and qPCR results of genes encoding proteins upregulated in all four conditions were only partial, which is not unexpected and highlights the importance of the proteomic approach in studying the cellular response to stress.

Among the proteins repeatedly shown to be upregulated, we observed known proteins involved in defense against oxidative stress, such as several glutathione transferases or peroxiredoxin (ACA1_027750), a member of the universal stress protein superfamily (ACA1_210670). More importantly, we identified four proteins that are upregulated regardless of the source of oxidative stress, and we believe that the function of these proteins deserves further rigorous investigation: a glutathione transferase (ACA1_099220), an NADPH-dependent FMN reductase (ACA1_175790), a phosphatase (ACA1_057530), and an oxidoreductase (ACA1_362830). The induction of glutathione transferase is consistent with the pluripotent role of the glutathione system in cellular detoxification and protection against oxidative stress. The role of NADPH-dependent FMN reductase in oxidative stress defense may be hypothesized due to NADPH being the principal reductant for the thioredoxin and glutathione systems. While the function of the identified phosphatase is difficult to propose due to the high variability of functions and substrates, AlphaFold structure prediction of the induced oxidoreductase revealed a close homology to human quinone oxidoreductase PIG3 (Figure S5). Contrary to the role in oxidative stress defense, this enzyme was shown to be associated with ROS generation in human and plant cells.^46,47^ Therefore, it would be beneficial in the future to biochemically characterize this protein, considering both its function in the Acanthamoeba oxidative stress response and the role of PIG3 in critical processes of human cells: response to DNA damage and p53-mediated apoptosis.^48^

Putting the results of our study in the context of previous research, we can conclude that Acanthamoeba employs a wide range of oxidative stress counteracting machineries, some of whose components are inducible at the transcriptomic/proteomic or enzymatic level. These include, in particular, the thioredoxin and glutathione systems, mitochondrial energy dissipating systems, and enzymes such as catalase or superoxide dismutase,^29,49,50^

Importantly, we have also identified an ABC transporter whose levels are increased upon incubation with rotenone and PEITC. Given that these two compounds are organic molecules and given the cellular localization of the transporter, it is very likely that this protein plays a critical role in drug efflux in *A. castellanii*. Interestingly, isothiocyanates (including PEITC) have been shown to interact with a number of ABC transporters.^51^ Therefore, further research in our laboratory is currently focused on studying its specificity and role in drug resistance.

To summarize, our study has provided complex insight into the oxidative stress response of the facultatively pathogenic amoeba *Acanthamoeba castellanii* and identified several key players in its defense system. This will enhance our understanding of the mechanisms by which *Acanthamoeba* can successfully evade the immune system and may also lead to the identification of new chemotherapeutic strategies, given the potential of redox-active antiparasitic agents.^52−54^

## Supporting Information Available

Table S1 – Proteomic analysis of *A. castellanii* in different ROS-inducing conditions. Table S2 – Table summarizing the proteomic analysis of the oxidative status of cysteines in different ROS-inducing conditions. Figure S1 – Growth curve of *A. castellanii*in the presence of different ROS-inducing agents. Figure S2 – Venn diagram showing the comparison of upregulated proteins in *A. castellanii* after the addition of ROS-inducing agents. Figure S3 – Oxyblot and SDS-PAGE showing protein distribution and damage in the presence of different ROS-inducing agents. Figure S4 – Western blot showing increased expression of thioredoxin reductase in cells exposed to H_2_O_2_. Figure S5 – Structural alignment of the *A. castellanii* oxidoreductase (PDF)
Gene AmoebaDB and their description (XLSX)
Peptides and their description (XLSX)
