# Supplementary material for: Molecular Mechanisms of Acanthamoeba castellanii Response to Different Sources of Oxidative Stress
Source: J Proteome Res. 2025 Jan 20;24(2):449–58. doi: 10.1021/acs.jproteome.4c00573 (PMC11812009; doi:10.1021/acs.jproteome.4c00573)
Supplement: Supplementary file 1 — pr4c00573_si_001.pdf [file pr4c00573_si_001.pdf]

Supporting Information (SI)

for

Molecular mechanisms of *Acanthamoeba castellanii* response to different sources of oxidative stress.

Kateřina Ženíšková[1], Pavel Stopka[2], Tania Martín-Pérez[3], Guillaume Chevreux[4], Maria Grechnikova[1], Eliška Drncová [1], Ronald Malych[1], Jan Mach[1], Julia Walochnik[3], Jean-Michel Camadro[4], Robert Sutak[1]*

[1]Department of Parasitology, Faculty of Science, Charles University, BIOCEV, Vestec, Czech Republic.

[2]Department of Zoology, Faculty of Science, Charles University, BIOCEV, Vestec, Czech Republic.

[3]Center for Pathophysiology, Infectiology and Immunology, Institute of Specific Prophylaxis and Tropical Medicine, Medical University of Vienna, Vienna, Austria.

[4]Université de Paris Cité, CNRS, Institut Jacques Monod, F -75013 Paris, France.

* Corresponding author: sutak@natur.cuni.cz

SI Table of Contents

Table S1 – Proteomic analysis of *A. castellanii* in diferent ROS inducing conditions

Table S2 - Table summarizing the proteomic analysis of the oxidative status of cysteines in diferent ROS inducing conditions

Figure S1- Growth curve of *A. castellan*ii in the presence of different ROS inducing agents

Figure S2 - Venn diagram showing the comparison of upregulated proteins in *A. castellanii* after addition of ROS inducing agents

Figure S3 – Oxyblot and SDS-PAGE showing protein distribution and damage in the presence of different ROS inducing agents

Figure S4 – Western blot showing increased expression of thioredoxin reductase in cells exposed to H_2O_2

Figure S5 - Structural alignment of the *A. castellanii* oxidoreductase

Figure S1

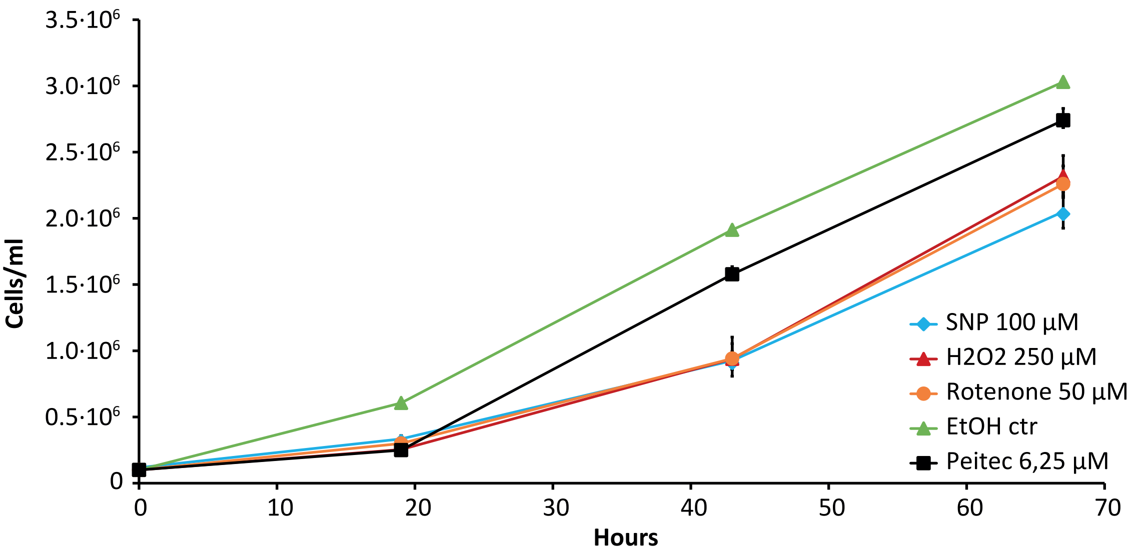

Growth curve of A. castellanii in the presence of different ROS-inducing agents. 100µM SNP (blue), 250µM H2O2 (red), 50µM rotenone (orange), 6.25µM PEITC (black), and 0.45% ethanol as a control (green).

Figure S2

[H2O2 UP] and [PEITC UP]:	
ACA1_181190	hypothetical protein
ACA1_116240	glutathione Stransferase, C-terminal domain containing protein
ACA1_201350	glutathione transferase family protein
ACA1_027750	peroxiredoxin, putative
ACA1_369270	rasrelated protein Rab-18-like, putative
ACA1_175640	alcohol dehydrogenase
ACA1_153660	DAD family protein
ACA1_071340	C2 domain containing protein
ACA1_075230	glutathione Stransferase domain containing protein
ACA1_142120	unspecified product
ACA1_184280	hypothetical protein
ACA1_150320	hypothetical protein
ACA1_202400	K+ channel protein
ACA1_089960	Dual specificity protein kinase
ACA1_150010	Nup85 Nucleoporin, putative
ACA1_142260	unspecified product
ACA1_231010	phosphoenolpyruvate carboxykinase
ACA1_077800	NAD(+) ADPribosyltransferase-3, putative

[PEITC UP] and [Rotenone UP]:	
ACA1_352460	ABC2 type transporter superfamily protein
ACA1_289660	churchill domain containing 1, putative
ACA1_290680	NHL repeat-containing protein
ACA1_330330	hypothetical protein
ACA1_080060	L-asparaginase I, putative
ACA1_046460	ABC2 type transporter superfamily protein
ACA1_324040	carrier superfamily protein
ACA1_187720	tetrahydrofolate dehydrogenase/cyclohydrolase, NAD(P)binding domain containing protein
ACA1_223920	saccharopine dehydrogenase (nad+, l-lysine forming), putative
ACA1_266390	BTB/POZ domain containing protein
ACA1_177560	proteasome subunit beta type7, putative
ACA1_396180	C2 domain containing protein
ACA1_040710	ATP-dependent metallopeptidase HflB subfamily protein

[PEITC UP] and [Rotenone UP] and [SNP UP]:	
ACA1_042320	hydroxyisourate hydrolase
ACA1_061250	parvulintype peptidyl-prolyl cis/trans isomerase
ACA1_210670	universal stress domain containing protein

[Rotenone UP] and [SNP UP]:	
ACA1_216040	protein kinase domain containing protein
ACA1_188240	metaxin 1, putative
ACA1_060130	BTB/POZ domain containing protein
ACA1_346030	CHCH domain containing protein
ACA1_074570	CHCH domain containing protein
ACA1_138520	putative phospholipase blike 2, putative
ACA1_077140	RNA recognition domain containing protein
ACA1_071970	Choline/Carnitine oacyltransferase superfamily protein
ACA1_271860	protein serine/threonine kinase
ACA1_138990	gammaadaptin 1, putative
ACA1_207510	hypothetical protein
ACA1_165530	alpha amylase, catalytic subfamily protein

[H2O2 UP] and [PEITC UP] and [Rotenone UP] and [SNP UP]:	
ACA1_362830	oxidoreductase, putative
ACA1_175790	NADPHdependent FMN reductase domain containing protein
ACA1_099220	glutathione transferase family protein
ACA1_057530	phosphatase, putative

[H2O2 UP] and [PEITC UP] and [Rotenone UP]:	
ACA1_279760	PWI domain containing protein

[H2O2 UP] and [Rotenone UP]:	
ACA1_075740	tetratricopeptide repeat domain containing protein
ACA1_139260	GTPbinding protein
ACA1_189400	PB1 domain containing protein
ACA1_214980	HAD hydrolase, family IA, variant 3 subfamily protein
ACA1_181960	autophagy protein, putative
ACA1_014390	hypothetical protein
ACA1_266780	ribosomal RNA large subunit methyltransferase J, putative
ACA1_058310	hypothetical protein

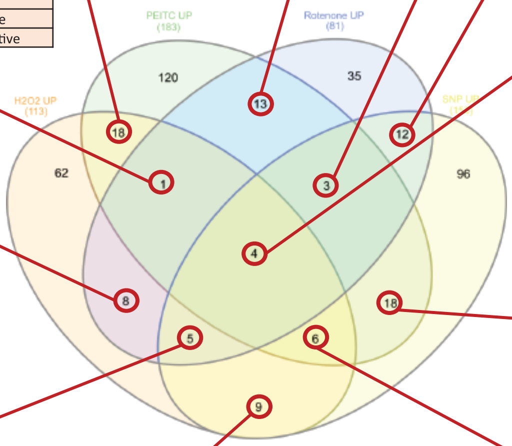

[PEITC UP] and [SNP UP]:	
ACA1_266790	glutathione Stransferase
ACA1_304310	cysteine protease
ACA1_066290	papain family cysteine protease subfamily protein
ACA1_033640	Ser/Thr phosphatase family protein
ACA1_376230	Hypothetical protein
ACA1_319220	cellular apoptosis susceptibility protein
ACA1_299380	zinc finger, NFX1-type containing 1, putative
ACA1_113860	acyloxyacyl hydrolase
ACA1_387070	Ltb4dh protein
ACA1_352400	glycosyl hydrolase domain containing protein
ACA1_400540	sphingosine hydroxylase
ACA1_364370	glutathione transferase family protein
ACA1_395480	copine subfamily protein
ACA1_014360	calcium/calmodulindependent protein kinase
ACA1_188500	CRAL/TRIO domain containing protein
ACA1_366110	MPPN (rrmlike) domain containing protein
ACA1_144880	hypothetical protein
ACA1_250300	carrier superfamily protein

[H2O2 UP] and [Rotenone UP] and [SNP UP]:	
ACA1_201160	RasGEF domain containing protein
ACA1_091450	Omethyltransferase
ACA1_265410	hypothetical protein
ACA1_163140	micropain, putative
ACA1_388310	micropain, putative

[H2O2 UP] and [SNP UP]:	
ACA1_149990	ubiquitin domain containing protein
ACA1_261480	carbohydrate kinase protein
ACA1_224370	Exportin 1like protein
ACA1_219490	2OGFe(II) oxygenase family oxidoreductase
ACA1_366990	Hypothetical protein
ACA1_063630	singlestranded DNA binding protein, putative
ACA1_381600	golgi family protein, putative
ACA1_103210	hypothetical protein
ACA1_043290	glutathione stransferase

[H2O2 UP] and [PEITC UP] and [SNP UP]:	
ACA1_076100	hypothetical protein
ACA1_364280	sporulation proteinrelated, putative
ACA1_385780	protein kinase domain containing protein
ACA1_292910	branched-chain alpha-KETO ACID decarboxylase E1 beta subunit, putative
ACA1_113950	hypothetical protein
ACA1_266210	hypothetical protein

Venn diagram showing the comparison of upregulated proteins in A. castellanii after addition of ROS-inducing agents. The diagram was generated based on proteomic analysis (Table S1).

Figure S3

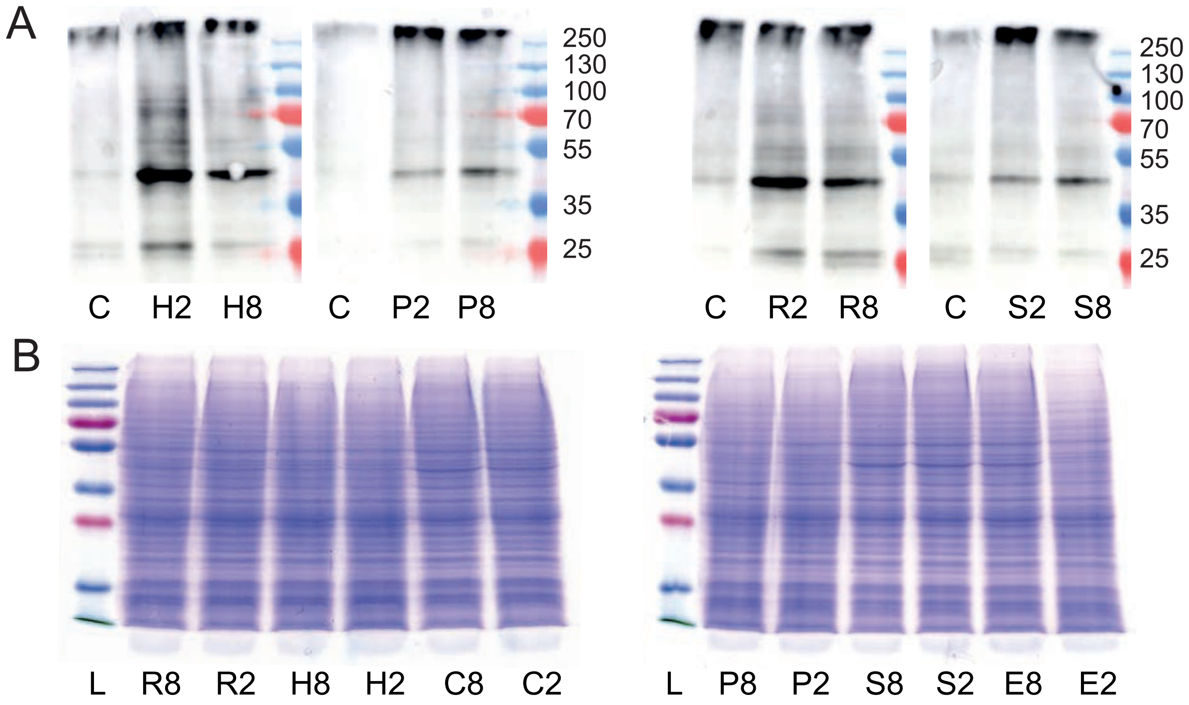

(A) Oxyblot showing the amount and distribution of damaged proteins during incubation of A. castellanii with different ROS-inducing agents. (B) Coomassie Blue stained SDS-PAGE is used as a loading control. C2 (2 hours; medium); C8 (8 hours; medium); H2 (2 hours, 250µM H2O2); H8 (8 hours, 250µM H2O2); P2 (2 hours, 6.25µM PEITC); P8 (8 hours, 6.25µM PEITC); R2 (2 hours, 50µM rotenone); R8 (8 hours, 50µM rotenone); S2 (2 hours, 100µM SNP); S8 (8 hours, 100µM SNP); E2 (2 hours; 0.45% ETOH); E8 (8 hours; 0.45% ETOH)

Figure S4

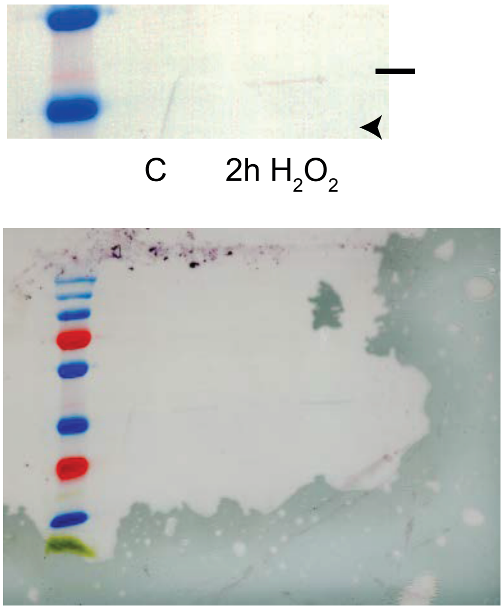

Increased expression of thioredoxin reductase (ACA1_398900) in cells exposed to 250 µM H2O2 for 2 hours using a specific antibody, confirming the result obtained by proteomic analysis. C – control with 0.45% ETOH; 2h H2O2 – cells incubated for 2 hours with 250µM H2O2. The entire membrane is displayed for the purpose of demonstrating data validity.

Figure S5

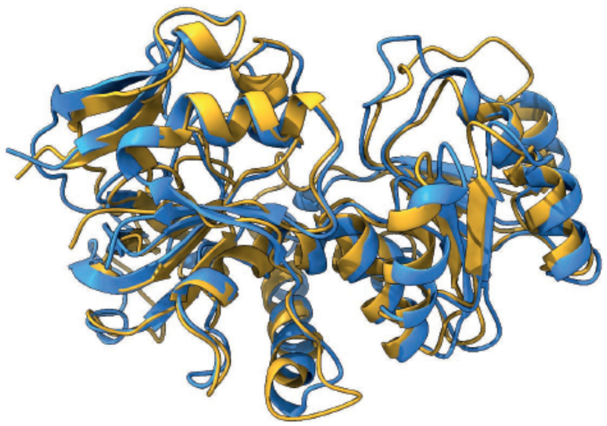

Structural alignment of the A. castellanii oxidoreductase (ACA1_362830, blue) predicted by Alphafold and the crystallographic structure of the Homo sapiens quinone oxidoreductase PIG3 (PDB 2J8Z, yellow). The structure prediction was performed by AlphaFold2 53, and the model was used as a query for a FoldSeek 54 search against the PDB structure database.
